# Obesity Is Associated with a Weakened Gingival Inflammatory Cytokine Response

**DOI:** 10.3390/medicina59122089

**Published:** 2023-11-28

**Authors:** Ahmed Khocht, Denise Bellinger, Leticia Lenoir, Crissy Irani, Gary Fraser

**Affiliations:** 1 Department of Periodontics, School of Dentistry, Loma Linda University, Loma Linda, CA 92350, USA; llenoir@llu.edu; 2 Department of Pathology and Human Anatomy, School of Medicine, Loma Linda University, Loma Linda, CA 92350, USA; dbellinger@llu.edu; 3 Institute for Community Partnerships, Loma Linda University Health, Loma Linda, CA 92354, USA; cirani@llu.edu; 4 Department of Preventive Medicine, School of Medicine, Loma Linda University, Loma Linda, CA 92350, USA; gfraser@llu.edu

**Keywords:** inflammatory cytokines, C-reactive protein, gingival fluid, obesity

## Abstract

*Background and Objectives*: An obesity-related elevated body mass index (BMI) across life is associated with chronic low-grade inflammation and increased levels of C-reactive protein (CRP) in blood. CRP is a marker and promoter of inflammation. The objectives of this study were to examine the effect of obesity on the relationship between peripheral and gingival CRP levels and to examine the effects of gingival CRP levels on gingival fluid inflammatory cytokines in periodontitis-resistant obese individuals. *Materials and Methods*: Thirty-nine participants in good periodontal health were recruited. Twenty patients were classified as lean and nineteen as obese based on their BMI levels. A thorough periodontal assessment was carried out. Gingival crevicular fluid (GCF) and blood samples were collected. Both GCF and blood samples were analyzed for interleukin-1β (IL-1β), interleukin-6 (IL-6), interleukin-8 (IL-8), tumor necrosis factor-α (TNF-α), interleukin-10 (IL-10), interleukin-17A (IL-17A), and CRP. *Results*: GCF CRP levels were significantly higher in the obese than in the lean individuals. No statistically significant differences were noted between the two groups in either GCF or blood in terms of any of the inflammatory cytokine levels. IL-17A was not detected in the GCF of most subjects in both groups. GCF CRP levels were positively associated with blood CRP levels, and the association tended to be stronger in the obese individuals. GCF CRP showed no associations with GCF IL-10 in both groups. Although GCF CRP levels were positively associated with multiple GCF inflammatory cytokines (e.g., IL-1β, IL-6, IL-8, and TNF-α) in all subjects, the associations tended to be weaker in the obese individuals (e.g., IL-1β, IL-6, and TNF-α). Furthermore, the levels of the GCF inflammatory cytokines IL-6 and TNF-α were decreased in the obese individuals. *Conclusions*: Obesity unfavorably influences the relationship between blood and GCF CRP levels and promotes increased CRP levels in GCF. Collectively, the findings suggest a weakened inflammatory cytokine response in the gingival tissues of obese individuals.

## 1. Introduction

Obesity is a chronic inflammatory disorder in which excessive fat accumulation presents a risk to health. In obesity, white fat tissue does not operate normally and does not expand to store excess energy. Enlarged visceral adipose tissue is infiltrated with proinflammatory immune cells (e.g., M1 macrophages and cytotoxic T cells), and it secretes excessive amounts of proinflammatory adipokines (e.g., adiponectin, leptin, visfatin, resistin, and chemerin) and cytokines (e.g., TNF-α, IL-1β, and IL-6) [1]. These adverse inflammatory signals induce a state of chronic systemic inflammation and increased levels of C-reactive protein (CRP) in blood [2].

CRP is an acute inflammatory protein and an important marker of systemic inflammation [3]. CRP is primarily synthesized in liver hepatocytes. Hepatocytes are the primary cells that exhibit the transcriptional activation of the CRP gene in response to elevated levels of inflammatory cytokines, specifically IL-6. Other cells, including macrophages, lymphocytes, endothelial cells, and adipocytes, can also synthesize CRP [3]. CRP is generated as a homopentameric protein known as native CRP (nCRP), which, at sites of inflammation, permanently splits into five distinct monomers known as monomeric CRP (mCRP) [3]. CRP was named for its response to the capsular (C)-polysaccharide of Pneumococcus [4]. CRP is not only a marker of infection and inflammation but also plays a protective role against bacterial infections through the activation of complement, the opsonization of pathogens, and the induction of inflammatory cytokines [3]. CRP is elevated in many inflammatory conditions, including obesity [2].

Periodontitis is a chronic inflammatory oral disease distinguished by gingival inflammation and the destruction of alveolar bone. Disproportionate host responses and bacterial dysbiosis are the two main etiological factors [5]. Failure in the resolution of gingival inflammation leads to destructive periodontal disease. In the pathogenesis of periodontitis, monocytes/macrophages play important roles in tissue destruction and repair. Their cytokine secretions contribute significantly to the inflammatory burden in the gingival tissues, they bridge innate immunity with adaptive immunity, and, finally, they enable the resolution of inflammation [6]. The major inflammatory cytokines involved in periodontitis include IL-1β [7,8], TNFα [9,10], IL-6 [11,12], IL-8 [13,14], IL-17A [5], and anti-inflammatory IL-10 [15,16].

Periodontal disease is prevalent among obese individuals [17,18], and the response to periodontal therapy is compromised [19]. Inflammation may provide a common link between periodontal disease and obesity [20,21]. Previously, it was shown that CRP levels are elevated in the gingival fluid of obese individuals [22]. Since CRP is a proinflammatory protein, we hypothesized that increased levels of CRP in the gingival tissues of obese individuals would promote gingival inflammation and may explain the increased susceptibility to periodontitis in obesity. Previous studies mainly focused on periodontally diseased individuals [22]. The effect of obesity on the periodontium without the confounding effect of periodontitis is not well understood. Obesity-related increased levels of CRP in gingival tissues may provide insightful information about how obesity affects periodontal health.

The aims of this study were to examine the effect of obesity on the relationship between peripheral and gingival CRP levels and to examine the association of gingival CRP levels with clinical periodontal measures and gingival fluid inflammatory cytokines in periodontally healthy obese individuals.

## 2. Materials and Methods

Recently, we described a distinct population of elderly individuals (>60 yrs.) who are resistant to periodontitis [23,24]. The recruitment and enrollment process for subjects has already been reported [23,24].

The Recruitment, Screening, and Enrollment of Subjects: A total of 136 individuals were interviewed over the phone to determine their eligibility. In total, 54 individuals agreed to participate, 39 of whom met the criteria for periodontal health (pocket depth ≤ 3 mm and bleeding on probing (BOP) < 10%) [25]. All subjects provided written informed consent. A history of diabetes, fewer than 15 teeth, pocket depth > 3 mm and BOP ≥ 10%, antibiotic treatment within the previous three months, dental cleaning within the previous three months, current tobacco use, non-English speaking, and limitations interfering with dental examinations were among the exclusion criteria.

A thorough medical history interview was conducted. The measures of height, weight, and BMI were recorded. Waist circumference was measured with a measuring tape placed around the abdomen at the top of the hip and at the level of the navel. A questionnaire about dietary preferences, educational background, income, dental care, and history of cigarette smoking was completed by all participants [23,24].

### 2.1. Clinical Assessments and Sample Collection

All dental exams were conducted by a single, experienced dental examiner (LL). The examiner was familiar with and trained in the use of the clinical assessments used in this study [23,24]. The plaque index (PI) and gingival index (GI) were recoded around all existing teeth [26]. Teeth with crowns, extensive restorations, partially erupted teeth, and third molars were not examined. Missing teeth and dental caries were also noted.

A standard periodontal probe (Michigan-O Probe Williams-labeled, Hu-Friedy, Chicago, IL, USA) was used for probing measurements. Probing depth (PD) was recorded at six sites per tooth: the mesiobuccal, buccal, distobuccal, mesiolingual, lingual, and distolingual sites. The position of the gingival margin (GM) in relation to the cementoenamel junction was recorded at the same six sites per tooth. The periodontal attachment level (AL) was calculated using the formula AL = PD + GM. BOP was also recorded.

Blood Samples: A blood sample was collected via a single vein puncture into serum-separating (red/black-topped) Vacutainer tubes at the scheduled clinic visit. Blood was allowed to clot at room temperature for 30 min; the clot was removed by centrifuging at 1000–2000× *g* for 15 min [27]. Serum was collected and aliquoted into 8 × 0.6 mL microcentrifuge tubes. Serum samples were frozen at −80 °C until analyzed.

GCF was collected with filter paper strips (Oraflow Inc., Smithtown, NY, USA). Three interproximal sites (PD ≤ 3 mm, no BOP, and no loss of attachment) were selected at random. Before collecting GCF samples, the chosen sites were isolated, supragingival plaque was removed, and the tooth surface was air-dried. After gently inserting the strip 1 to 2 mm into the gingival crevice, it was left in place for 30 s. Blood-contaminated strips were discarded. Sample volume was estimated using a Periotron 8000 (Oraflow Inc., Smithtown, NY, USA). The 3 GCF strips collected from each subject were placed in a single microcentrifuge tube. The tube was sealed, put on ice, and then frozen at −80 °C until the laboratory analysis was performed. All clinical evaluations and sample collections were performed on the same day.

### 2.2. Laboratory Analyses

The GCF and serum samples were thawed and processed for analysis prior to running cytokine assays as per the standard protocol. The GCF and serum samples were analyzed for IL-1β, IL-6, IL-8, IL-10, IL-17A, TNF-α, and CRP.

The Elution of GCF: the three strips from each vial were immersed in 1 mL of elution buffer consisting of PBS containing 0.1% Triton X-100 and 0.1% bovine serum albumin (BSA) [28]. The elution process was performed overnight in a refrigerator [28]. To determine the percentage of recovery, a set of controls for each biomarker (in which a known concentration of each biomarker was added to the filter papers) was utilized to simulate the elution of the biological components under investigation from the filter paper strips. Every biomarker had a recovery rate of at least 98%. Standard curves were generated in the same elution buffer, thus preserving the integrity of the matrix.

CRP concentrations were determined using enzyme-linked immunosorbent assay (ELISA) kits from AssayPro (St. Charles, MO, USA). Each sample was assayed in duplicate in accordance with the manufacturers’ instructions [27], and the data are expressed as ng/mL. The minimal detectable concentration was 100 pg/mL. IL-1β, IL-6, IL-8, IL-10, IL-17A, and TNF-α levels were determined simultaneously using a human cytokine/chemokine Milliplex Luminex magnetic bead multiplexed panel (EMD Millipore, Billerica, MA, USA) coated with specific antibodies (Milliplex MAP Kit), and they are expressed as pg/mL. The samples were run in duplicate as directed by the manufacturer. Fluorescence was read using a Luminex 200 (Millipore). Analytes were normalized to the total protein concentration determined using a Bradford assay (using the elution buffer as the baseline), and the data are expressed as pg/mL.

### 2.3. Statistical Analysis

This is a convenience sample of a previously described periodontitis-resistant cohort [23,24]. A total of 20 subjects were lean (BMI < 25), 11 subjects were overweight (BMI 25–<30), and 8 subjects were obese (BMI ≥ 30). Overweight and obese subjects were combined into one group for data analysis. CRP, cytokine levels, and clinical periodontal measures were all positively skewed. For statistical analyses, non-parametric tests were employed. The Wilcoxon rank-sum test for continuous measurements and the Pearson chi-square test for categorical variables were used to identify significant differences between groups.

Zero-order correlations between blood CRP, GCF CRP, periodontal measures, and GCF inflammatory cytokines were determined with Pearson correlation analyses. We used rank regression analyses adjusted for age, gender, and race to examine the associations of the measures of obesity (BMI and waist size) with peripheral CRP levels, peripheral CRP levels with GCF CRP levels, GCF CRP with clinical measures of gingival health, and GCF inflammatory cytokines. A type 1 error rate of 5% was used.

## 3. Results

The demographics and clinical data of this cohort were previously reported and are summarized in Table 1 [24].

Nineteen subjects were classified as overweight/obese, while twenty subjects were classified as lean (BMI of ≥25). All subjects were medically healthy and non-smokers. The two groups were statistically comparable on age, sex, race, blood pressure readings, the number of missing teeth, PI, GI, BOP, PD, and AL. As predicted, the obese group had considerably greater weight, waist size, and BMI scores. The subjects in both groups satisfied the criterion for periodontal health. PI tended to be higher in the obese subjects, even though it was statistically comparable between the two groups. All subjects reported that they practiced oral hygiene at least once a day and received preventive dental care on average twice a year. The subjects’ blood pressure readings fell within the normal ranges for their age. Systolic blood pressure was somewhat higher in the obese individuals.

In Table 2, we compare blood and GCF CRP and inflammatory cytokine levels between the lean and obese individuals. For GCF measures (Table 2a), the univariate analyses showed that the two groups were statistically comparable on all measures, except for GCF CRP, with levels being significantly higher in the obese individuals than in the lean individuals. Despite the lack of statistical significance, IL-6 and TNF-α levels tended to be higher in the lean than in the obese individuals; IL-8 tended to be higher in the obese than in the lean individuals. GCF IL-17A was detected at low levels in only two subjects (one lean subject and one obese subject) and was excluded from analysis. The lack of detectible GCF IL-17A in most subjects suggests that IL-17A activity is absent in healthy gingival tissues. For blood measures (Table 2b), both CRP and inflammatory cytokine levels were statistically comparable between the obese and lean subjects.

**Figure 1:**
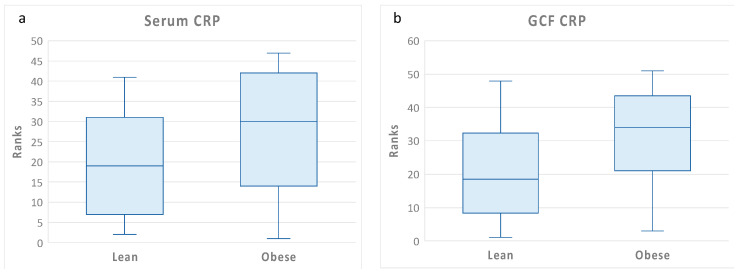
Blood and gingival fluid CRP levels according to obesity status. Box plots of rank-transformed CRP levels according to obesity status. The bottom and top sides of each box represent the lower and upper quartiles, respectively. The line inside the box represents the median. The bottom and top whiskers represent the minimum and maximum values, respectively. (**a**) Blood CRP: Wilcoxon rank-sum test (untransformed actual data) showed no statistically significant difference between the two groups (Z = 1.18, *p* < 0.23). ANCOVA (rank-transformed data) adjusting for age, gender, and race confirmed the univariate analysis (F(4,32) = 2.06, *p* = 0.16). (**b**) GCF CRP: Wilcoxon rank-sum test (untransformed actual data) showed that GCF CRP levels were significantly higher in obese than in lean individuals (Z = −2.02, *p* < 0.04). ANCOVA (rank-transformed data) adjusting for age, gender, and race confirmed the univariate analysis (F(4,37) = 4.77, *p* = 0.03).

The differences in GCF and blood CRP levels between the lean and obese individuals are illustrated in Figure 1. Although blood CRP levels tended to be higher in the obese than in the lean individuals, the differences were not statistically significant. Blood CRP levels showed a high variance among the obese individuals, which may explain the lack of statistical significance between the two groups. By contrast, GCF levels were significantly higher in the obese than in the lean individuals.

**Figure 2:**
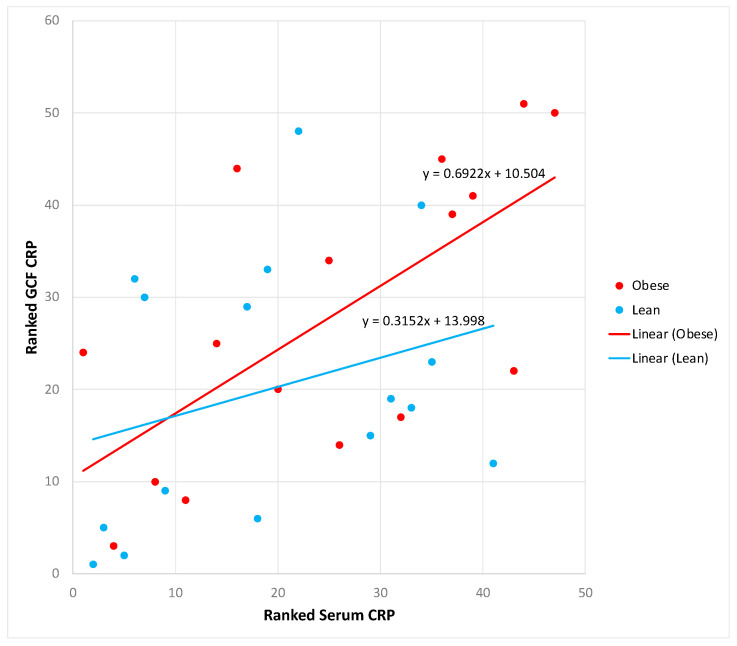
Scatter plots and regression lines of serum CRP (independent variable) versus GCF CRP (dependent variable) according to group, with unadjusted rank transformed data. Slope equations are indicated. Pairwise slope difference test: Difference (obese – lean) = 0.37, *p* = 0.27. Pearson correlation coefficients for obese individuals: r = 0.63, *p* = 0.008, and for lean individuals: r = 0.40, *p* = 0.12.

We then examined the association between blood and gingival CRP levels. The Pearson correlation analysis indicated a significant correlation between blood and GCF CRP levels in all subjects combined (r = 0.52, *p* = 0.001). Figure 2 presents the regression line slopes of the association of blood with GCF CRP according to obesity status. Although the line slop of the obese group was steeper than that of the lean group, the difference was not statistically significant.

Blood cytokine measures showed no statistically significant associations with obesity measures (BMI and waist size), blood pressure measures, or GCF cytokine measures (data not presented).

Next, we investigated the association of GCF CRP levels and oral hygiene (PI scores) with measures of gingival health and GCF inflammatory cytokines. Zero-order correlation analyses showed significant positive associations between GCF CRP levels with multiple measures of gingival health and GCF inflammatory cytokines in both the lean and obese individuals (Table 3a). The correlation coefficients for IL-1β, IL-6, and TNF-α tended to be stronger in the lean individuals; by contrast, the correlation coefficient for IL-8 tended to be stronger in the obese individuals (Table 3a). These data suggest that GCF CRP levels may be involved in the promotion of inflammation in gingival tissues. IL-10 showed no association with GCF CRP in both subject groups, and the GCF IL-1β association with GCF CRP in the obese subjects lacked statistical significance (Table 3a). In Table 3b, we compare the association of PI scores with measures of gingival health and GCF inflammatory cytokines between the lean and obese individuals. In both groups, PI was significantly associated with BOP, but the association was weaker in the obese individuals. No statistically significant correlations were noted between PI and inflammatory cytokines. The Pearson correlation coefficients between PI and inflammatory cytokines were poor in both groups and tended to be even weaker in the obese than in the lean individuals.

**Figure 3:**
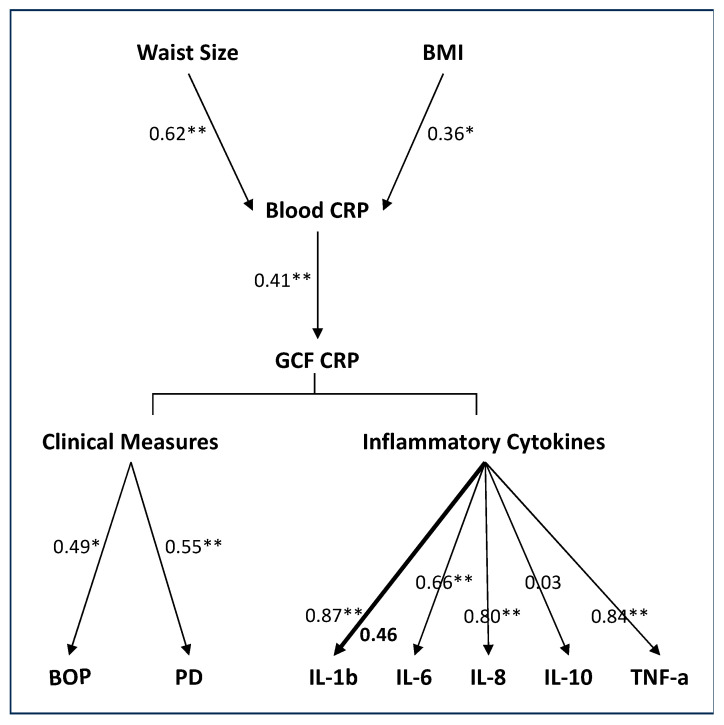
Flow diagram outlining the hypothesized association sequence of measures of obesity (BMI and waist size) with peripheral CRP levels, peripheral CRP levels with GCF CRP levels, GCF CRP with clinical measures of gingival health, and GCF inflammatory cytokines. Rank regression models adjusted for age, race, and gender were used for prediction of dependent variables. Data are presented as standardized beta weight. When the strength of association was comparable between the two groups, a light arrow with a single value is presented. When the strength of association differed between the two groups, a heavy arrow with two values is presented; values of obese individuals are bolded. *p* < 0.05 *, *p* < 0.01 **.

Finally, in a series of rank regression models adjusted for age, race, and gender, we further examined the association of measures of obesity (BMI and waist size) with peripheral CRP levels, peripheral CRP levels with GCF CRP levels, GCF CRP with clinical measures of gingival health, and GCF inflammatory cytokines. Figure 3 outlines the noted associations between the various factors assessed in the multivariate analyses. As expected, both waist size and BMI measures were positively associated with serum CRP levels. Waist size measures showed a stronger association with serum CRP levels than BMI measures.

In the adjusted models, the association between blood and GCF CRP levels remained significant. Again, the strength of the association was statistically comparable between the lean and the obese individuals. The multivariate analyses also confirmed the positive associations of GCF CRP with clinical measures of gingival health (BOP and PD) and the inflammatory cytokines IL-6, IL-8, and TNF-α; the strengths of the associations for these measures were statistically comparable between the two groups. The association of GCF CRP with IL-1β showed a group interaction, and the association was significant only in the lean individuals. The non-significant association between GCF CRP and IL-1β in the obese individuals confirms the univariate findings.

**Figure 4:**
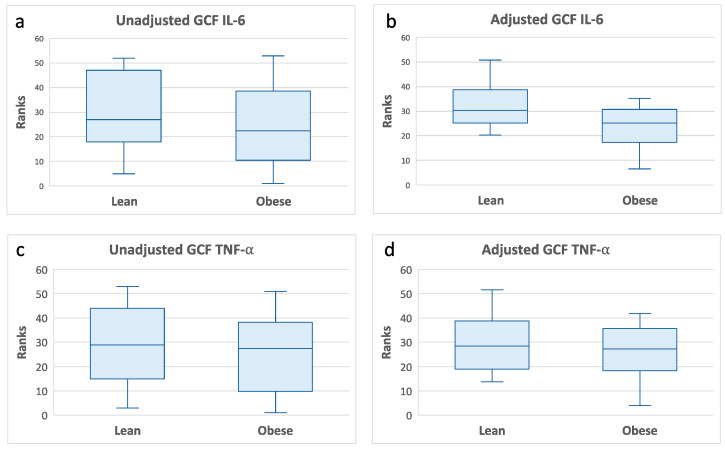
GCF IL-6 and TNF-α levels according to obesity status. Box plots of rank-transformed cytokine levels according to obesity status. The bottom and top sides of each box represent the lower and upper quartiles, respectively. The line inside the box represents the median. The bottom and top whiskers represent the minimum and maximum values, respectively. IL-6: (**a**) Wilcoxon rank-sum test (untransformed actual data) showed no statistically significant difference between the two groups (Z = −0.93, *p* < 0.35). (**b**) ANCOVA of rank transformed data adjusting for PI and GCF CRP levels indicated that GCF IL-6 levels in lean individuals were significantly higher than in obese individuals (F(3,34) = 10.68, *p* = 0.002). TNF-α: (**c**) Wilcoxon rank-sum test (untransformed actual data) showed no statistically significant difference between the two groups (Z = −0.16, *p* < 0.86). (**d**) ANCOVA of rank-transformed data adjusting for PI and GCF CRP levels indicated that GCF TNF-α levels in lean individuals were significantly higher than in obese individuals (F(3,34) = 8.45, *p* = 0.006).

Additional multivariate analyses adjusting for oral hygiene (PI scores) did not alter the previously noted associations between GCF CRP with clinical measures of gingival health and the inflammatory cytokines IL-1β, IL-6, IL-8, and TNF-α (data not presented). PI showed an association with BOP (standardized beta = 0.52, *p* = 0.0006) independent from GCF CRP levels. In addition, the multivariate analyses also showed that IL-6 and TNF-α levels were significantly lower in the obese than in the lean individuals (Figure 4).

## 4. Discussion

The aims of this study were to examine the effect of obesity on the association between peripheral blood and GCF CRP levels and to examine the association between GCF CRP and GCF inflammatory cytokines in periodontally healthy obese individuals without the confounding effect of periodontal disease. The data showed that blood CRP levels positively correlated with GCF CRP levels in all subjects, and the association tended to be stronger in the obese individuals. GCF CRP levels were significantly higher in the obese than in the lean individuals. Although GCF CRP levels were positively associated with multiple GCF inflammatory cytokines (e.g., IL-1β, IL-6, and TNF-α) in all subjects, the associations tended to be weaker in the obese individuals. Furthermore, the levels of the GCF inflammatory cytokines IL-6 and TNF-α were decreased in the obese individuals. Collectively, these findings suggest a weakened inflammatory cytokine response to increased levels of CRP in the gingival tissues of obese individuals.

The Adventist Health Study-2 (AHS-2) cohort, which consists of over 96,000 Seventh-Day Adventists in the US and Canada, was used to recruit study participants [29,30]. Health-conscious Seventh-Day Adventists build on good health practices by avoiding meat and emphasizing a plant-based diet rich in whole grains, legumes, and nuts. To increase power and supplement the limited number of individuals available, overweight and obese subjects were combined into a single group. The average BMI of the obese participants in this study was 32, indicating low-level obesity. The clinical measures, CRP levels, and cytokine levels of the obese and overweight participants were statistically similar. The health-conscious behavior and good systemic health of this cohort may explain the lack of differences in systemic inflammatory biomarker levels between the lean and obese individuals.

The primary site of CRP production is the liver [3]. The CRP levels in blood reflect the presence of inflammation [3]. Our data showed a significant correlation between blood and GCF CRP levels in all subjects, suggesting that blood CRP contributes to GCF CRP levels. Contrary to our findings, a previous study reported a lack of correlation between blood and the GCF levels of CRP in periodontitis subjects [31]. It is possible that gingival inflammation in periodontitis may mask the association between blood and GCF CRP levels. Our data also showed that obesity has an unfavorable influence on the association between blood and GCF CRP levels; the association was more pronounced in the obese individuals. Despite the variability in blood CRP levels in this cohort, the obese individuals tended to have higher blood CRP levels. This may explain the stronger association between blood and CRP levels in the obese individuals. However, the difference in the strength of the association was not statistically significant. Again, the variance in blood CRP levels may also explain the lack of statistical significance.

In the current study, GCF CRP levels were significantly elevated in the obese individuals. These data support those of previous studies showing elevated GCF CRP in obese individuals [32] and expand on previous reports by including periodontitis-resistant elderly individuals. GCF is a transudate of serum and thus includes both serum constituents and locally produced molecules. Blood is the primary contributor of CRP in GCF [33]. In this cohort, there was variance in blood CRP levels among the obese subjects. Yet, despite the blood CRP variance, gingival fluid demonstrated significantly elevated levels of CRP in the obese individuals. These findings suggest the possibility of local CRP production in the gingival tissues of obese individuals. Although a previous study did not show CRP mRNA in the gingival tissues [33], gingival CRP production was not examined in obesity. Possible local cells that may contribute to gingival CRP production may include lymphocytes [34], macrophages [35], and epithelial cells [36].

CRP is not only a marker of inflammation but also plays an active role in the inflammatory process [3]. Our data showed significant associations between GCF CRP levels and IL-1β, IL-6, IL-8, and TNF-α cytokine measures.

IL-1β is a proinflammatory cytokine with angiogenic activity that promotes blood vessel formation in inflamed tissues [37]. IL-1β levels are elevated in inflamed gingival tissues [38]. Gingival epithelial cells produce IL-1β in response to microbial pathogens [39]. Activated macrophages and other inflammatory cells release TNF-α, a significant cytokine that promotes inflammation [10]. TNFα is elevated in the GCF of individuals with gingival inflammation [38,40] and helps the inflammatory front advance further into gingival connective tissue. IL-6 is a multifunctional proinflammatory cytokine primarily produced by T cells. Its main role is the final differentiation of B lymphocytes into plasma cells, which is associated with periodontal tissue damage [12].

Previous research showed that elevated CRP levels in atheroma induce the production of IL-1β, IL-6, and TNF-α by macrophages [41]. A similar effect may occur in gingival tissues, where increased levels of CRP may promote inflammatory cytokine production from local immune cells. Conversely, an increased production of inflammatory cytokines in the gingival tissues may induce the local transcription of CRP [42,43,44].

Our data showed that the association of GCF CRP with IL-1β was statistically nonsignificant in the obese individuals. Furthermore, the multivariate analyses showed that GCF IL-6 and TNF-α levels were decreased in the obese individuals. These data suggest a dysregulated inflammatory cytokine response to increased levels of CRP in the gingival tissues of obese individuals. The main contributors of inflammatory cytokines in the gingiva are macrophages [45]. In an animal model, the infiltration of macrophages was significantly decreased in the gingival tissues of obese mice [46]. Furthermore, obese mice with periodontitis showed a weakened macrophage inflammatory response with a reduced expression of the NLRP3 signal pathway [46]. These obesity-related alterations in macrophage number and function in the gingival tissues may lead to innate immune dysfunction and adversely affect cytokine production. Human studies examining gingival macrophage activity in obesity are lacking.

The chemokine IL-8 is mostly secreted by macrophages and monocytes. The recruitment and activation of neutrophils are its primary roles [13]. The neutrophils and monocytes recruited by IL-8 release copious inflammatory cytokines, such as IL-1β and TNF-α. CRP plays a role in atherosclerosis via enhanced IL-8 production and an increased expression of IL-8 mRNA [47]. CRP promotes IL-8 production via the activation of the ERK, p38 MAPK, and JNK pathways [47]. In our study, IL-8 was the only cytokine that tended to show a strong association with GCF CRP in the obese subjects, suggesting that IL-8 production in gingival tissues is not weakened by obesity. Unlike IL-1β, IL-6, and TNF-α, which are primarily produced by cells of the immune system, such as monocytes, macrophages, and T-helper cells, IL-8 is produced by numerous other cell types, including inflammatory cells, as well as keratinocytes, fibroblasts, and endothelial cells. Although the infiltration and function of immune cells in the gingival tissues of obese individuals may be compromised, other cells may compensate for immune cell deficiency and produce IL-8 in the gingival tissues of obese individuals.

The anti-inflammatory cytokine IL-10 inhibits the activation of immune cells. IL-10 levels tend to be inversely associated with pocket depth [13]. Our data showed no association between GCF IL-10 and GCF CRP. CRP decreases IL-10 production in immune cells via the inhibition of cyclic AMP production [48]. The combination of obesity-related monocyte dysfunction and the suppressing effect of CRP on IL-10 secretion may explain the noted lack of association between GCF CRP and IL-10 in our results.

Despite this study’s limitations, which include the limited sample size and participants’ low-grade obesity, relevant associations were noted between the obesity-related increase in GCF CRP levels and measures of periodontal health and GCF inflammatory cytokines, which further our understanding of how obesity may impact periodontal health. Future studies examining local cytokine production in relation to immune cell infiltration in the gingival tissues of obese individuals could further enhance our understanding of how obesity impacts gingival immune/inflammatory responses and identify common inflammatory pathways that can be pharmacologically targeted to improve the response to therapy.

## 5. Conclusions

Obesity unfavorably influences the relationship between blood and GCF CRP levels and promotes increased CRP levels in GCF. Our findings were contrary to our hypothesis. Despite the obesity-related increase in GCF CRP levels, the clinical measures of gingival inflammation and GCF inflammatory cytokine levels were not significantly increased. The association between GCF CRP and GCF IL-1β levels in the obese individuals lacked statistical significance. Furthermore, the levels of the GCF inflammatory cytokines IL-6 and TNF-α were decreased in the obese individuals. Collectively, these findings suggest a weakened inflammatory cytokine response in the gingival tissues of obese individuals.

## Figures and Tables

**Table 1:** Demographics and clinical measures according to obesity status.

Variable	Lean (BMI < 25)	Obese (BMI ≥ 25)	*p*-Value
a. Demographics			
Age (years)	69.1 (2.18)	71.42 (1.71)	0.55
% Male	45	47	0.77
% Black	30	37	0.65
b. Clinical Measures			
Weight (lbs.)	153.2 (5.49)	183.3 (8.18)	0.009
Waist (cm)	87.91 (83.54)	102.5 (96.94)	0.0003
BMI	22.26 (0.35)	29.65 (0.73)	0.0001
Missing teeth	1.95 (0.58)	2.52 (0.60)	0.51
PI	0.44 (0.06)	0.63 (0.08)	0.06
GI	0.58 (0.06)	0.58 (0.06)	0.83
GCF volume	2.06 (0.23)	1.93 (0.26)	0.59
BOP%	4.28 (1.14)	5.84 (1.30)	0.32
PD mm	1.85 (0.06)	1.98 (0.04)	0.16
AL mm	1.32 (0.21)	1.34 (0.19)	0.63

Data are presented as mean (SE) or percentage. *p* value gives the probability that the groups differ in either Wilcoxon test using ranks or chi-square test for proportions. AL, attachment level; BMI, body mass index; BOP, bleeding on probing; GI, gingival index; PD, probing depth.

**Table 2:** CRP and cytokine measures according to obesity status.

Variable	Lean (BMI < 25)	Obese (BMI ≥ 25)	*p*-Value
**(a) Gingival Fluid**			
GCF CRP (ng/mL)	0.03 (0–0.08)	0.07 (0.03–0.54)	0.04
IL-1β (pg/mL)	1.41 (0.27–9.99)	2.58 (1.54–5.7)	0.17
IL-6 (pg/mL)	0 (0–1.91)	0 (0–0.57)	0.34
IL-8 (pg/mL)	57.59 (22.24–307.80)	103.68 (58.78–282.08)	0.16
IL-10 (pg/mL)	5 (0–5)	0 (0–5)	0.21
TNF-α (pg/mL)	0 (0–1.49)	0 (0–0.68)	0.85
**(b) Blood (Serum)**			
CRP (µg/mL)	7.6 (2.4–18.2)	9.8 (4.42–29.5)	0.22
IL-1β (pg/mL)	1.84 (1.26–2.14)	1.2 (0.78–3.64)	0.52
IL-6 (pg/mL)	5 (2.86–8.67)	5.06 (2.92–11.9)	1
IL-8 (pg/mL)	10.56 (5.9–21.62)	10.6 (6.52–13.54)	0.93
IL-10 (pg/mL)	16.2 (5.12–23.36)	11.98 (5.38–37.14)	0.89
IL-17A (pg/mL)	12.47 (9.07–25.84)	11.96 (8.8–27.52)	1
TNF-α (pg/mL)	9.7 (7.98–11.29)	10.38 (8.2–14.02)	0.27

Data reported as median (25th percentile to 75th percentile). *p* value gives the probability that the groups differ in Wilcoxon test using ranks. GCF IL-17A was not detected in most subjects (data not presented).

**Table 3:** Pearson correlation analyses examining the associations of GCF CRP and PI with clinical measures of gingival health and GCF inflammatory cytokines according to obesity status.

	Lean		Obese	
Variable	r	*p*-Value	r	*p*-Value
**(a) GCF CRP Correlations**				
BOP%	0.45	0.06	0.56	0.01
PD (average)	0.57	0.01	0.32	0.2
IL-1β (pg/mL)	0.64	0.003	0.36	0.1
IL-6 (pg/mL)	0.80	0.0001	0.52	0.02
IL-8 (pg/mL)	0.48	0.03	0.82	0.0001
IL-10 (pg/mL)	0.09	0.7	0.09	0.7
TNF-α (pg/mL)	0.83	0.0001	0.73	0.0004
**(b) PI Correlations**				
BOP%	0.59	0.005	0.48	0.03
PD-ave	0.17	0.45	0.20	0.40
IL-1β (pg/mL)	0.28	0.23	0.06	0.79
IL-6 (pg/mL)	0.15	0.51	−0.01	0.94
IL-8 (pg/mL)	0.15	0.50	−0.16	0.49
IL-10 (pg/mL)	0.12	0.60	0.01	0.95
TNFα (pg/mL)	0.18	0.43	−0.12	0.61

Data presented as Pearson correlation coefficients.

## Data Availability

The data published in this study are available upon reasonable request from the first author.
